# LRP1 Mediates Endocytosis Activity and Is a Potential Therapeutic Target in Osteoarthritis

**DOI:** 10.1111/os.70035

**Published:** 2025-04-02

**Authors:** Yuangang Wu, Kaibo Sun, Mingyang Li, Yang Yang, Yuan Liu, Limin Wu, Yang Ding, Yi Zeng, Bin Shen

**Affiliations:** ^1^ Orthopedic Research Institute and Department of Orthopedics Surgery West China Hospital, Sichuan University Chengdu China

**Keywords:** endocytosis, low‐density lipoprotein receptor‐related protein 1, osteoarthritis, therapeutic target

## Abstract

Osteoarthritis (OA) is a degenerative disease characterized by cartilage abrasion and pain, affecting millions globally. However, current treatments focus on symptom management rather than modifying disease development. Recent studies have indicated that low‐density lipoprotein receptor‐related protein 1 (LRP1) is associated with maintaining cartilage homeostasis through its involvement in endocytosis and signaling pathways. LRP1 facilitates the removal of extracellular matrix (ECM)‐degrading enzymes, including a disintegrin and metalloproteinase with thrombospondin motifs (ADAMTSs) and matrix metalloproteinases (MMPs), thereby protecting against excessive cartilage breakdown. However, OA cartilage shows increased shedding of LRP1, leading to reduced endocytic capacity and elevated levels of these enzymes, contributing to accelerated ECM breakdown. LRP1 is also involved in key signaling pathways, such as Wnt/β‐catenin, transforming growth factor‐beta (TGF‐β), and nuclear factor‐kappa B (NF‐κB), which regulate processes like chondrocyte proliferation, apoptosis, differentiation, and autophagy. Dysregulation of these pathways, combined with impaired LRP1‐mediated endocytosis, fosters a catabolic environment in osteoarthritic cartilage. Emerging therapies targeting LRP1, such as gene interventions, exosome‐based therapies, and small‐molecule modulators, show potential in restoring LRP1 function, reducing cartilage degradation, and promoting joint repair. This review emphasizes the significance of LRP1 in the development of OA and explores its potential as a therapeutic target for creating disease‐modifying strategies to maintain joint integrity and enhance patient well‐being.

## Graphical abstract

(A) The risk factors associated with OA development. (B) Exosomes derived from TNF‐α stimulate upregulation of LRP1 levels in IPFP‐MSC‐derived exosomes, thereby exerting chondroprotective effects. (C) The regulation of endocytosis by LRP1, which impacts chondrocyte proliferation and apoptosis. (D) LRP1 mediates cartilage matrix homeostasis through interactions with specific compounds and growth factors. (E) By facilitating the endocytosis and degradation of multiple matrix‐degrading enzymes, LRP1 regulates chondrocyte function and ECM metabolism in cartilage. (F) LRP1 represents a promising therapeutic target for subchondral bone remodeling in OA. OA, osteoarthritis; LRP1, low‐density lipoprotein receptor‐related protein 1; TNF‐α, tumor necrosis factor‐alpha; IPFP‐MSCs: infrapatellar fat pad‐mesenchymal stem cells; ECM, extracellular matrix. MMP‐13: matrix metalloproteinase‐13; ERK: extracellular signal‐regulated kinase; TIMP‐3: tissue inhibitor of metalloproteinases‐3; ADAMTS‐5: a disintegrin and metalloproteinase with thrombospondin motif 5; PAR‐2: protease‐activated receptor 2; TGF‐β1: transforming growth factor beta1; Wnt5b: Wingless‐type MMTV integration site family, member 5B.

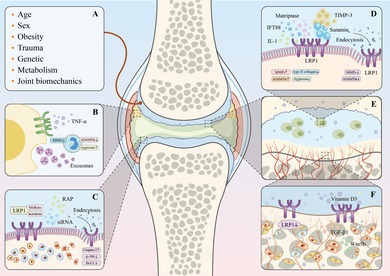

## 1 Introduction

Osteoarthritis (OA) is a chronic and progressive joint disorder characterized by the degeneration of articular cartilage [1, 2]. As the most prevalent type of arthritis, OA affects more than 250 million individuals globally and is a leading cause of disability and diminished quality of life, especially in older populations [3, 4, 5, 6]. Despite its complex and multifactorial pathophysiology [7, 8, 9, 10, 11, 12], there are no available disease‐modifying therapies capable of halting or reversing cartilage damage [13, 14]. Consequently, this underscores the urgent need for innovative therapeutic strategies aimed at improving patient outcomes.

Low‐density lipoprotein receptor‐related protein 1 (LRP1) is a crucial member of the LDL receptor family, widely recognized for its involvement in mediating endocytosis and regulating cellular signaling pathways [15, 16, 17]. This receptor family includes several structurally similar proteins, such as the low‐density lipoprotein receptor (LDLR), very low‐density lipoprotein receptor (VLDLR), and LRP1, all of which play vital roles in lipid transport and tissue homeostasis [17, 18, 19, 20, 21]. Among them, LRP1 is a multifunctional receptor expressed in a variety of tissues, contributing significantly to extracellular molecule clearance and the regulation of cellular functions [22, 23, 24]. The dysregulation of LRP1 has been linked to the onset of various diseases, including cardiovascular conditions and neurodegenerative disorders [16, 25, 26].

Research has highlighted the critical role of LRP1 in cartilage biology, particularly in regulating the balance between anabolic and catabolic processes within the extracellular matrix (ECM) of cartilage [27, 28, 29, 30]. Highly expressed in chondrocytes, LRP1 acts as an endocytic receptor for ECM‐degrading enzymes, including matrix metalloproteinases (MMPs) and a disintegrin and metalloproteinase with thrombospondin motifs (ADAMTSs) [31, 32, 33, 34]. By mediating the endocytosis and degradation of these enzymes, LRP1 helps to limit excessive ECM breakdown, thereby maintaining cartilage integrity [35, 36, 37]. In osteoarthritic cartilage, however, increased shedding of LRP1 leads to reduced endocytic capacity, resulting in elevated extracellular levels of MMPs and ADAMTSs, which in turn accelerate cartilage degradation [33, 38]. On the contrary, studies have shown that inhibition of LRP1 shedding can restore its endocytic function, reduce ECM degradation, and potentially reverse the progression of OA [38, 39]. Moreover, LRP1 engages with multiple signaling pathways, including Wnt/β‐catenin, nuclear factor‐kappa B (NF‐κB), and transforming growth factor beta (TGF‐β), all of which play essential roles in chondrocyte survival and differentiation [40, 41, 42]. Dysregulation of these pathways, combined with impaired LRP1‐mediated endocytosis, contributes to the catabolic environment characteristic of osteoarthritic cartilage [40, 42]. Thus, targeting LRP1 pathways represents a promising therapeutic strategy for OA.

This review aims to provide a comprehensive analysis of the role of LRP1 in the development and progression of OA, highlighting its involvement in regulating endocytosis activity, ECM turnover, and cellular signaling.

## 2 Literature Screening Methodology

To ensure a comprehensive review of the role of LRP1 in OA, we conducted a structured literature search strategy.

### 2.1 Literature Search Strategy

A systematic search was performed in the following databases: PubMed, Web of Science, Embase, and the Google Academic is also used to search for additional literature. The search strategy included combinations of the following keywords: (“LRP1” OR “low‐density lipoprotein receptor‐related protein 1”) AND (“osteoarthritis” OR “cartilage degradation” OR “endocytosis” OR “chondrocyte signaling” OR “cartilage homeostasis”).

### 2.2 Inclusion and Exclusion Criteria

Studies were screened based on predefined inclusion and exclusion criteria:

Inclusion criteria: (1) Original research articles, reviews, or meta‐analyses published in peer‐reviewed journals. (2) Studies investigating the function of LRP1 in OA or cartilage homeostasis. (3) Articles in English published between 2000 and 2024. (4) Studies utilizing in vitro, in vivo, or clinical models to assess LRP1‐mediated pathways.

Exclusion criteria: (1) Studies lacking relevance to OA or cartilage‐related processes. (2) Case reports, editorials, conference abstracts, or opinion pieces. (3) Studies without sufficient experimental or clinical data.

### 2.3 Screening Results

**FIGURE 1:**
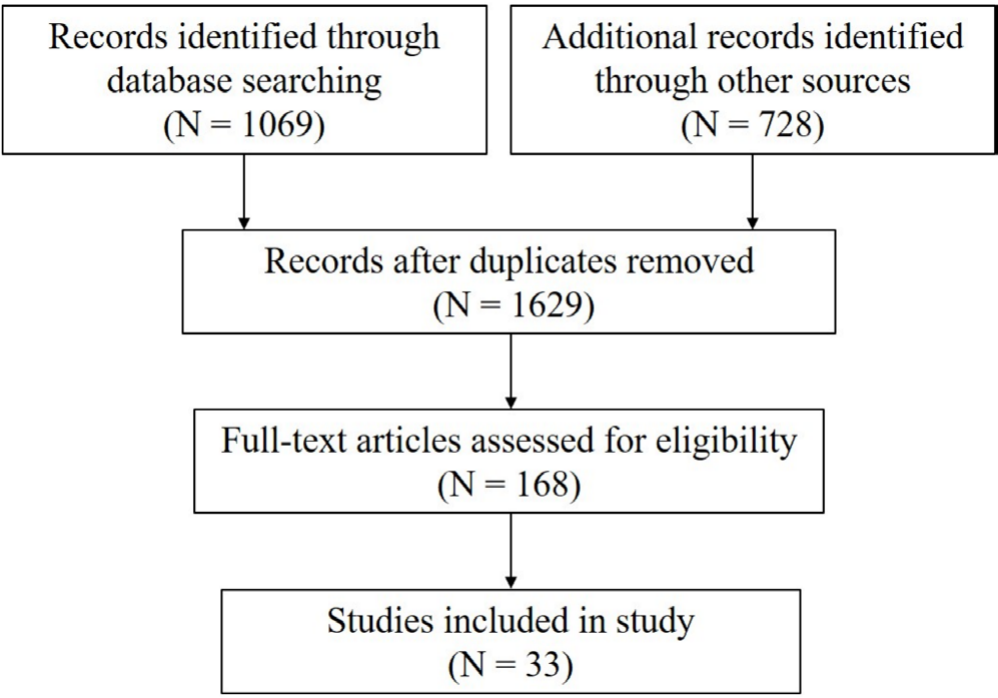
The flowchart in this study.

Two independent reviewers screened titles and abstracts based on relevance to LRP1 and OA. Relevant data, including study type, methodology, key findings, and experimental models, were extracted. A total of 1797 articles were identified from database searches. After removing duplicates of 1629 articles, 168 articles remained for title and abstract screening. Following this phase, 168 articles were selected for full‐text review. Finally, 33 studies met the inclusion criteria and were included in the review. The flowchart was presented in Figure 1.

## 3 Structure and Function of LRP1

**FIGURE 2:**
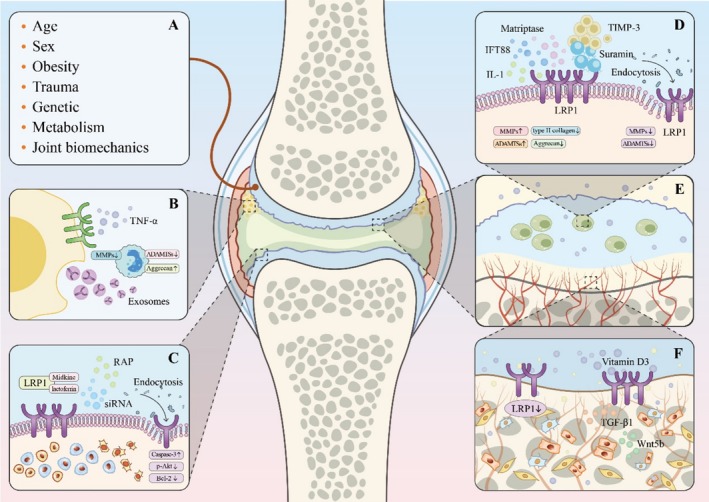
Pathogenesis related to LRP1 in OA. (A) The risk factors associated with OA development. (B) Exosomes derived from TNF‐α stimulate upregulation of LRP1 levels in IPFP‐MSC‐derived exosomes, thereby exerting chondroprotective effects. (C) The regulation of endocytosis by LRP1, which impacts chondrocyte proliferation and apoptosis. (D) LRP1 mediates cartilage matrix homeostasis through interactions with specific compounds and growth factors. (E) By facilitating the endocytosis and degradation of multiple matrix‐degrading enzymes, LRP1 regulates chondrocyte function and ECM metabolism in cartilage. (F) LRP1 represents a promising therapeutic target for subchondral bone remodeling in OA. ADAMTS‐5: A disintegrin and metalloproteinase with thrombospondin motif 5;ECM, extracellular matrix; ERK: Extracellular signal‐regulated kinase; IPFP‐MSCs: Infrapatellar fat pad‐mesenchymal stem cells; LRP1, low‐density lipoprotein receptor‐related protein 1; MMP‐13: Matrix metalloproteinase‐13; OA, osteoarthritis; PAR‐2: Protease‐activated receptor 2; TGF‐β1: Transforming growth factor beta1; TIMP‐3: Tissue inhibitor of metalloproteinases‐3; TNF‐α, tumor necrosis factor‐alpha; Wnt5b: Wingless‐type MMTV integration site family, member 5B.

LRP1 is a versatile and multifunctional receptor essential for mediating endocytosis and cellular signaling [43, 44]. It is initially synthesized as a 600‐kDa precursor protein, which undergoes proteolytic processing in the Golgi apparatus, yielding two noncovalently associated subunits: a 515‐kDa heavy chain (α‐subunit) and an 85‐kDa light chain (β‐subunit) [45, 46]. The α‐subunit features four distinct clusters of ligand‐binding repeats, which are essential for interacting with a diverse array of ligands such as proteases, growth factors, and ECM proteins. These binding clusters are interspersed with epidermal growth factor (EGF) precursor homology domains and six YWTD repeats, which form β‐propeller structures that enhance the specificity of ligand interactions [47, 48] The β‐subunit consists of a transmembrane domain and a short cytoplasmic tail that includes various motifs, such as two NPXY motifs, a YXXL motif, and two dileucine motifs. These motifs play crucial roles in intracellular trafficking and signaling pathways [49, 50]. The NPXY motifs act as binding sites for cytoplasmic adaptor proteins, facilitating LRP1's involvement in diverse intracellular signaling pathways. Meanwhile, the YXXL motif is essential for directing LRP1 to clathrin‐coated vesicles during the endocytic process [51, 52]. Collectively, these structural features allow LRP1 to function as a versatile endocytic receptor capable of regulating the extracellular environment by internalizing and clearing numerous ligands (Figure 2).

**FIGURE 3:**
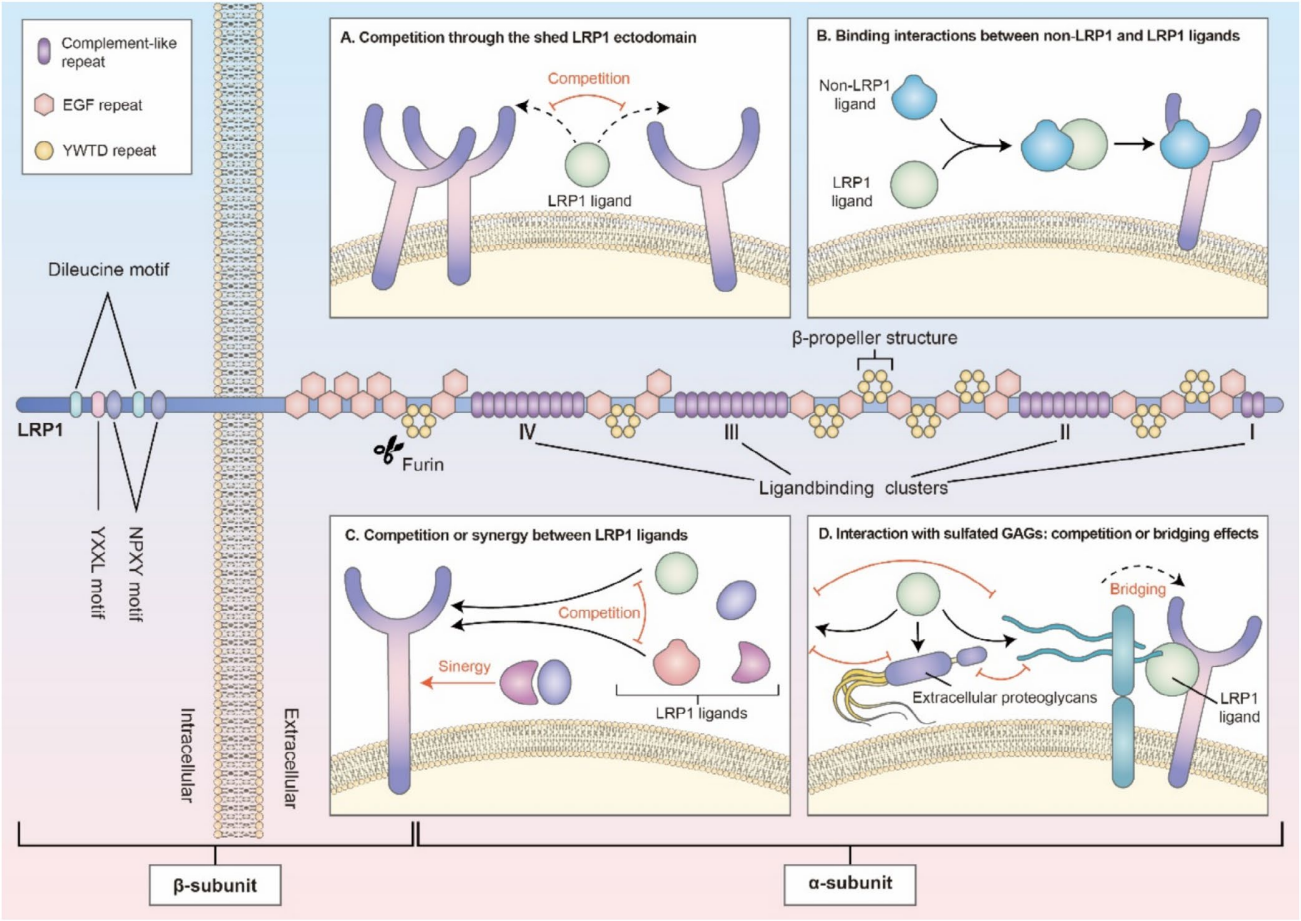
The protein structure and functional domains of LRP1. LRP1 undergoes cleavage by furin within the Golgi apparatus, producing two noncovalently associated subunits: (A) 515‐kDa α subunit and an 85‐kDa β subunit. The α subunit features clusters of cysteine‐rich complement‐type repeats, known as ligand‐binding repeats, arranged into four groups (I–IV). These clusters mediate interactions with numerous LRP1 ligands and are interspersed with EGF precursor homology regions and six YWTD repeats that form a β‐propeller structure. The β subunit includes two NPXY motifs, a YXXL motif, and two dileucine motifs. A. Competition through the shed LRP1 ectodomain; (B) Binding interactions between non‐LRP1 and LRP1 ligands; (C) Competition or synergy between LRP1 ligands. (D) Interaction with sulfated GAGs: Competition or bridging effects. Abbreviation: LRP1, low‐density lipoprotein receptor‐related protein 1; EGF, epidermal growth factor; GAG, glycosaminoglycan.

Recent research has revealed that LRP1 is broadly expressed across multiple tissues, including the liver, brain, and cartilage, with particularly high levels in chondrocytes [53, 54, 55, 56]. In OA, LRP1 plays a pivotal role in cartilage homeostasis through the endocytosis and degradation of ECM‐degrading enzymes, thereby preserving the balance between ECM synthesis and degradation [57, 58, 59] (Figure 3). Consequently, understanding the structure–function relationship of LRP1 is essential for elucidating its role in OA pathogenesis.

## 4 LRP1 Involvement in Cartilage Matrix Homeostasis in OA

### 4.1 Proliferation and Apoptosis

Chondrocyte proliferation and apoptosis are essential processes that regulate cartilage homeostasis and integrity [60]. Research has highlighted the critical role of LRP1 in controlling chondrocyte proliferation and apoptosis within cartilage [39, 41, 61]. For instance, midkine (MK) has been reported to enhance chondrocyte proliferation by activating the LRP1–nucleolin signaling pathway. A study by Deng et al. demonstrated that recombinant human MK stimulates chondrocyte proliferation in vitro by interacting with LRP1, which acts as a receptor to facilitate MK endocytosis [61]. Following endocytosis, MK forms a complex with nucleolin, activating the K‐Ras signaling cascade and leading to extracellular signal‐regulated kinase (ERK) 1/2 pathway activation and cyclin D1 upregulation [61]. This process ultimately promotes chondrocyte proliferation, highlighting the therapeutic potential of MK in cartilage regeneration and the treatment of OA pathology.

Similarly, Yamamoto et al. utilized a top‐down proteomic approach to explore the LRP1 ligandome in chondrocytes, focusing on how LRP1‐mediated endocytosis regulates chondrocyte survival and function [39]. Their findings indicate that inhibition of LRP1 or its ligand interactions leads to chondrocyte detachment and cell death, underscoring the importance of LRP1 in maintaining cartilage cell survival. Furthermore, Yang et al. examined the impact of LRP1 knockdown on tumor necrosis factor‐alpha (TNF‐α)‐induced apoptosis in chondrocytes [41]. Their findings revealed that silencing LRP1 elevated TNF‐α‐induced expression of matrix metalloproteinase‐13 (MMP‐13), resulting in decreased synthesis of type II collagen and increased chondrocyte apoptosis. Additionally, LRP1 knockdown reduced the levels of antiapoptotic proteins such as phospho‐Akt and Bcl‐2, while upregulating proapoptotic markers like caspase‐3 [41]. Together, these findings underscore the essential function of LRP1 in cartilage biology and highlight promising strategies for modulating chondrocyte proliferation and apoptosis to manage OA.

### 4.2 MMPs

Dysregulation of MMP‐13 expression or its clearance can result in excessive collagen degradation, leading to cartilage breakdown and OA progression [62, 63]. Thus, understanding the mechanisms that regulate MMP‐13 activity, particularly through interactions with receptors for LRP1, is crucial for developing targeted therapies for OA (Table 1). Raggatt et al. explored the involvement of LRP1 in mediating the internalization and signaling of MMP‐13 in chondrocytes. Their study revealed that LRP1 specifically binds to and facilitates the internalization of MMP‐13. Notably, this internalization process also triggered the activation of the ERK1/2 signaling pathway, suggesting that LRP1 plays a dual role, contributing not only to proteolysis but also to intracellular signaling [29]. However, the activation of ERK1/2 was found to be independent of LRP1, suggesting that MMP‐13 might exert additional effects on chondrocyte function through alternative pathways [29].

**TABLE 1:** The Cartilage Matrix Homeostasis Interaction With LRP1 in OA.

Name	Cell/tissue type	Mechanism	Function summary	References
ADAMTS‐5	Adipose‐derived stromal cells	Suppresses glucose uptake, reduces proteoglycan turnover	ADAMTS5 knockout promotes glucose uptake and ECM synthesis; protective against cartilage degradation	[34]
ADAMTS‐5	Cartilage	Regulates aggrecan degradation; binds LRP1 via spacer domain	Elevated activity in OA due to impaired LRP1‐mediated clearance	[35]
ADAMTS‐5	Articular cartilage	Regulated by monoclonal antibodies; rapid endocytosis via LRP1	Critical for cartilage ECM degradation; antibody blocks LRP1 interaction	[59]
MMP‐13	OA chondrocytes	Regulated by LRP1‐mediated endocytosis	Promotes cartilage matrix degradation; involved in OA progression	[27]
MMP‐13	Rabbit chondrocytes	ERK1/2 phosphorylation independent of LRP1	Induces collagen degradation; regulates chondrocyte phenotype	[29]
MMP‐13	Chondrocytes	Coendocytosed with TIMP‐3 via LRP1; cleaves collagen fibrils	Key collagenase in OA; activity tightly regulated by LRP1	[57]
Matriptase	OA cartilage	Activates MMPs and PAR‐2	Promotes aggrecan degradation; inhibition reduces OA cartilage damage	[64]
TIMP‐3	Chondrocytes, fibroblasts	LRP1‐mediated endocytosis; retained by soluble LRP1	Inhibits ECM‐degrading enzymes; increases ECM stability	[33]
TIMP‐3	Chondrocytes, ECM	Regulates ECM turnover; Binds to sulfated glycosaminoglycans	Inhibits ADAMTS‐5; protects against cartilage degradation	[36]
TIMP‐3	ECM, chondrocytes	Binds LRP1 for endocytosis; inhibits ADAMTS‐5 and MMP‐13	Prevents cartilage degradation; reduced activity leads to ECM breakdown	[38]
TIMP‐3 Variants	Human Chondrocytes	Increased ECM binding; resistant to LRP1‐mediated endocytosis	Prolonged cartilage protection; inhibits cartilage degradation in vitro and in vivo	[56]

Abbreviations: ADAMTS‐5, a disintegrin and metalloproteinase with thrombospondin motif 5; ECM, extracellular matrix; ERK, extracellular signal‐regulated kinase; LRP1, low‐density lipoprotein receptor‐related protein 1; MMP‐13, matrix metalloproteinase‐13; OA, osteoarthritis; PAR‐2, protease‐activated receptor 2; TIMP‐3, tissue inhibitor of metalloproteinases‐3.

In contrast, increased shedding of LRP1 in osteoarthritic cartilage impairs the clearance of MMP‐13, resulting in enhanced extracellular collagen degradation. Blocking LRP1 shedding with specific monoclonal antibodies targeting LRP1 sheddases, such as ADAM‐17 and MMP‐14, has been demonstrated to restore the endocytic function of LRP1. This restoration effectively reduces the degradation of aggrecan and collagen in OA cartilage, mitigating matrix destruction [38]. Additionally, the regulatory role of LRP1 in maintaining ECM stability by modulating tissue inhibitor of metalloproteinases‐3 (TIMP‐3) activity has been highlighted by Scilabra et al. [33]. In osteoarthritic cartilage, LRP1‐mediated endocytosis of TIMP‐3 is impaired due to increased LRP1 shedding. The shed soluble form of LRP1 (sLRP1) retains the capacity to bind TIMP‐3 but lacks the ability to internalize it, thereby extending TIMP‐3's inhibitory effect on MMP‐13 activity [33]. These findings highlight LRP1 as a critical regulator of MMP‐13 activity in cartilage, suggesting that targeting LRP1 sheddases to enhance its endocytic function represents a promising therapeutic approach to reduce ECM degradation in OA.

### 4.3 ADAMTSs

The enzymatic activity of ADAMTS‐4 and ADAMTS‐5 is pivotal in the pathogenesis of OA, as their excessive degradation of aggrecan contributes significantly to cartilage breakdown [65, 66]. Regulating ADAMTS activity through key receptors, such as LRP1, is therefore critical for identifying novel therapeutic targets aimed at reducing cartilage degradation in OA [35, 37]. Yamamoto et al. investigated how LRP1 facilitates the endocytic clearance of ADAMTS‐4 in human chondrocytes [37]. They discovered that the cysteine‐rich and spacer domains of ADAMTS‐4 are essential for its binding to LRP1, with the thrombospondin‐1 (TS1) domain also playing a supportive role in this interaction. Interestingly, ADAMTS‐5 was found to inhibit the endocytosis of ADAMTS‐4 through competitive binding, while ADAMTS‐4 did not exhibit the same effect on ADAMTS‐5. This competition arises from the higher binding affinity of ADAMTS‐5 to LRP1, suggesting that under pathological conditions, ADAMTS‐5 may dominate LRP1‐mediated clearance, resulting in the accumulation of ADAMTS‐4 and exacerbated aggrecan degradation in osteoarthritic cartilage [37]. This study sheds light on the differential interactions between ADAMTS‐4 and ADAMTS‐5 with LRP1, offering new perspectives on the mechanisms of cartilage degradation.

Furthermore, Yamamoto et al. revealed that LRP1 effectively mediates the endocytic clearance of ADAMTS‐5, limiting its aggrecanase activity in normal cartilage [35]. However, in osteoarthritic cartilage, this endocytic mechanism is impaired, leading to an accumulation of ADAMTS‐5 and increased aggrecan degradation. Studies using receptor‐associated protein (RAP), an antagonist of LRP1 ligand binding, along with siRNA‐mediated gene silencing, have demonstrated that inhibiting LRP1 activity leads to a marked increase in aggrecan degradation mediated by ADAMTS‐5 [35]. These results highlight the potential for developing therapeutic strategies that target the interaction between ADAMTS enzymes and LRP1 to mitigate excessive aggrecan breakdown in osteoarthritic cartilage.

### 4.4 Aggrecan

Cleavage of aggrecan by aggrecanases, such as ADAMTS‐5, represents an early event in OA, facilitating subsequent collagen degradation by MMPs [67, 68]. The primary cilium protein intraflagellar transport protein 88 (IFT88) has been shown to regulate aggrecanase activity in chondrocytes by facilitating LRP1‐mediated endocytosis [69]. Disruption of IFT88 in chondrocytes led to increased aggrecanase activity and decreased LRP1‐mediated endocytosis, resulting in elevated extracellular protease levels [69].

Ismail et al. investigated how interleukin‐1 (IL‐1) influences aggrecan degradation, revealing that IL‐1 activates the JNK‐2 signaling pathway, which enhances aggrecanase activity while impairing LRP1‐mediated endocytosis of aggrecanase [55]. In parallel, Wilkinson et al. investigated the function of matriptase, a type II transmembrane serine protease, in facilitating the release of aggrecan from osteoarthritic cartilage [64]. Matriptase was found to induce aggrecanolysis by promoting metalloproteinase activity, including the shedding of LRP1. Inhibition of matriptase reduced cartilage damage and aggrecan degradation, highlighting the potential of targeting matriptase activity to protect against ECM breakdown in OA [64]. These findings highlight the critical need to preserve LRP1 function and regulate aggrecanase activity to protect cartilage integrity.

### 4.5 Collagenases

Collagenases, such as MMP‐1 and MMP‐13, play a vital role in breaking down fibrillar collagens, including type II collagen, which serves as the primary structural element of cartilage [70, 71]. In OA, these collagenases contribute significantly to cartilage ECM degradation. Kawata et al. demonstrated that silencing LRP1 in chondrocytic cells resulted in elevated levels of MMP‐13, a key collagenase involved in the degradation of type II collagen [31]. These findings imply that LRP1 suppresses collagenase expression by participating in signaling pathways that regulate chondrocyte differentiation and ECM turnover. Building on this, Wang et al. examined the influence of glycosylation on LRP1 function and its effect on collagenase activity [72]. Their research investigated *N*‐glycosylation patterns in chondrocytes derived from induced pluripotent stem cells (iPSCs) and revealed that variations in LRP1 glycosylation alter its capacity to bind and internalize ECM‐degrading enzymes, including collagenases. Variations in glycosylation were associated with changes in collagenase activity within osteoarthritic cartilage, indicating that post‐translational modifications of LRP1 may play a critical role in shaping its regulatory function in maintaining cartilage homeostasis [72].

### 4.6 TIMP‐3

TIMP‐3 is essential for preserving cartilage integrity by inhibiting MMPs and aggrecan‐degrading enzymes, both of which contribute to OA pathogenesis [73]. Unlike other TIMP family members, TIMP‐3 uniquely binds to the ECM, making it a critical regulator of ECM turnover and tissue stability [36]. Doherty et al. investigated the effects of engineered TIMP‐3 variants resistant to endocytosis via LRP1 on cartilage protection [56]. The researchers developed TIMP‐3 mutants, including TIMP‐3 K26A/K45A and K42A/K110A, with diminished binding affinity to LRP1. These mutants exhibited an extended extracellular presence and enhanced ability to inhibit metalloproteinase‐driven cartilage degradation compared to the wild‐type protein. Furthermore, suramin, a compound that binds to TIMP‐3, prevents its endocytosis via LRP1, leading to elevated extracellular TIMP‐3 levels and a subsequent reduction in cartilage degradation.

Green et al. expanded their research to examine suramin analogs and their potential to elevate TIMP‐3 levels by blocking LRP1‐mediated endocytosis [74]. They found that specific suramin analogs with distinct structural features were particularly effective in maintaining elevated TIMP‐3 levels in cartilage, making them promising candidates for OA treatment [74]. These findings present a significant step forward in developing disease‐modifying therapies for OA, targeting metalloproteinase activity at the level of post‐translational regulation of TIMP‐3.

## 5 LRP1 Regulated OA via Diverse Mechanisms

### 5.1 Wnt/β‐Catenin Signaling Pathway

The Wnt/β‐catenin signaling pathway plays an essential role in overseeing cell proliferation, differentiation, and apoptosis in skeletal tissues. Its activation has been implicated in the upregulation of MMP expression and the stimulation of osteophyte formation, both of which are key contributors to OA pathogenesis [75, 76]. Therefore, regulating Wnt/β‐catenin signaling, particularly through key receptors like LRP1, is crucial for developing new therapeutic approaches to mitigate OA progression [77]. LRP1, in association with the chaperone CRELD2, plays a significant role in modulating Wnt signaling during skeletal development [78]. CRELD2 facilitates LRP1 transport to the cell surface, regulating Wnt4 expression in chondrocytes via TGF‐β1 signaling. This highlights the importance of LRP1 in maintaining skeletal cell differentiation and suggests a novel mechanism by which LRP1 modulates noncanonical Wnt signaling, thereby contributing to endochondral ossification and bone integrity [78].

Additionally, research has investigated the involvement of genes within the Wnt/β‐catenin pathway and their contributions to knee OA [40]. These studies suggest that the function of LRP1 in the Wnt/β‐catenin pathway is embedded in a sophisticated genetic network that drives OA progression by modulating gene interactions and cellular mechanisms critical for maintaining cartilage health [40]. Hu et al. demonstrated the involvement of LRP1 in modulating the Wnt/β‐catenin pathway in osteoarthritic osteoblasts (Obs). The study identified two populations of OA Obs based on their osteocalcin response to vitamin D3 stimulation, suggesting heterogeneity in their regulatory capabilities of Wnt signaling [42]. Although LRP1 expression remained unchanged between these populations, other pathway‐related factors, such as Wnt5b and DKK2, were differentially expressed. This indicates that LRP1 collaborates with other elements of the Wnt pathway to regulate osteoblast phenotypes in the context of OA [42]. These observations highlight promising therapeutic targets that focus on modulating LRP1 and Wnt/β‐catenin signaling to mitigate cartilage degradation and promote bone integrity in OA (Table 2).

**TABLE 2:** The Mediated Mechanism of LRP1 in OA.

Name	Cell/tissue type	Mechanism	Function summary	References
LRP1	Chondrocytes	Regulates Wnt/β‐catenin signaling; mediates CCN2 endocytosis	Promotes chondrocyte differentiation; prevents hypertrophy during endochondral ossification; facilitates ECM remodeling	[28]
LRP1	Chondrocytes	Regulates Wnt/β‐catenin signaling	Maintains mature chondrocyte phenotype; controls ECM remodeling	[31]
LRP1	Chondrocytes	Mediates CCN2 transcytosis via endocytosis; regulates distribution of CCN2 under hypoxic conditions	Maintains ECM homeostasis; promotes chondrocyte differentiation; facilitates CCN2 transport in prehypertrophic zones	[32]
LRP1	Chondrocytes	Mediates endocytosis of ADAMTS‐4 and ADAMTS‐5 via cysteine‐rich and spacer domains	Regulates extracellular matrix homeostasis by controlling aggrecanase activity; impaired in OA	[37]
LRP1	Human chondrocytes and osteoblasts	Genetic polymorphisms affecting Wnt signaling and matrix remodeling	Regulates OA progression via cartilage and bone remodeling; significant LRP1 interactions in Wnt pathway	[40]
LRP1	Chondrocytes	Regulates TNF‐α‐induced NF‐κB signaling; modulates ECM proteolysis	Inhibits apoptosis and inflammation in OA; knockdown increases MMP‐13 activity	[41]
LRP1	Subchondral bone in early OA	Differential expression affects ECM remodeling	Potential early intervention targets for OA treatment	[79]
LRP1 Ligandome	Human cartilage	Identifies > 50 ligands, including growth factors and ECM proteins	Regulates chondrocyte survival and ECM homeostasis; impaired in OA	[39]
LRP1 SNPs	Articular cartilage	Modulation of WNT/β‐catenin signaling; linked to genetic risk of osteoarthritis	Decreases osteoarthritis risk; affects cartilage matrix remodeling	[77]
LRP1 variants	Triradiate cartilage, acetabulum	Suppresses autophagy and increases β‐catenin signaling	Impaired cartilage development leading to developmental dysplasia of the hip	[80]

Abbreviations: ADAMTS‐4, a disintegrin and metalloproteinase with thrombospondin motif 4; ADAMTS‐5, a disintegrin and metalloproteinase with thrombospondin motif 5; ECM, extracellular matrix; ERK, extracellular signal‐regulated kinase; LRP1, low‐density lipoprotein receptor‐related protein 1; MMP‐13, matrix metalloproteinase‐13; NF‐κB, nuclear factor‐kappa B; OA, osteoarthritis; TNF‐α, tumor necrosis factor‐alpha; Wnt/β‐catenin, wingless/beta‐catenin.

### 5.2 ERK Signaling Pathway

The ERK signaling pathway is pivotal in maintaining cartilage homeostasis and facilitating repair by orchestrating the balance among chondrocyte proliferation, differentiation, and apoptosis [81, 82]. LRP1 has been identified as a key regulator of ERK signaling in chondrocytes, impacting various aspects of cartilage biology, including matrix metabolism, cell survival, and inflammation [29, 30].

One study explored the interaction between LRP1 and lactoferrin in human articular chondrocytes and its impact on ERK1/2 signaling [30]. Their results showed that lactoferrin binding to LRP1 rapidly activated ERK1/2, thereby stimulating chondrocyte proliferation. The activation of ERK signaling by LRP1 highlights a key mechanism through which chondrocyte growth is promoted in response to extracellular stimuli, with potential implications for cartilage repair. Deng et al. explored the function of MK in stimulating chondrocyte proliferation through the LRP1–ERK signaling pathway. Their findings revealed that recombinant human MK binds to LRP1, triggering ERK1/2 activation, which in turn upregulates cyclin D1 expression to facilitate cell cycle progression [61].

Interestingly, Raggatt et al. provided intriguing insights into the effect of MMP‐13 on ERK signaling in rabbit chondrocytes, showing that MMP‐13 binding triggered ERK1/2 phosphorylation through a mechanism independent of LRP1‐mediated internalization [29]. This suggests that MMP‐13 may activate ERK signaling through an alternative mechanism (Table 3).

**TABLE 3:** The Compound and Growth Factor‐Mediated LRP1 in OA.

Name	Cell/tissue type	Mechanism	Function summary	Dose/concentration	References
CRELD2	Chondrocytes and osteoblasts	Modulates WNT4 signaling via LRP1; promotes TGF‐β1 signaling	Regulates skeletal differentiation and growth plate ossification; deficiency leads to skeletal abnormalities	100 nM SMARTpool ON‐TARGETplus siRNA for CRELD2	[78]
Interleukin‐1	Human chondrocytes	LRP1 shedding affects aggrecanase endocytosis	Promotes aggrecan degradation in osteoarthritis; inhibits ECM repair	Interleukin‐1 at 50 ng/mL	[55]
Lactoferrin	Human articular chondrocytes	Activates ERK1/2 signaling; LRP1 signaling	Promotes chondrocyte proliferation; enhances MMPs activity; inhibits aggrecan synthesis	Lactoferrin at 50 μg/mL	[30]
Midkine	Human and rat articular chondrocytes	MK‐LRP1‐nucleolin signaling; activates ERK1/2 and cyclin D1 expression	Promotes chondrocyte proliferation and cell cycle progression; potential OA therapy	Midkine at 3 μg/mL	[61]
Suramin	Human osteoarthritic cartilage	Inhibits TIMP‐3 endocytosis via LRP1; increases extracellular TIMP‐3 levels	Reduces cartilage degradation by inhibiting MMPs and ADAMTSs activity; provides chondroprotection	Suramin at 200 μg/mL	[58]
Suramin Analogs	Human osteoarthritic cartilage	Blocks TIMP‐3 endocytosis via LRP1; increases ECM‐bound TIMP‐3 levels	Protects cartilage against OA degradation; enhances TIMP‐3 longevity	Suramin Analogs at 100 μg/mL	[74]
Vitamin D3	Osteoblasts	Modulates TGF‐β1 and Wnt5b signaling pathways	Two OA osteoblast populations identified based on vitamin D3‐induced osteocalcin expression	Vitamin D3 at 50 nM	[42]

Abbreviations: ADAMTSs, a disintegrin and metalloproteinase with thrombospondin motifs; CRELD2, cysteine‐rich with epidermal growth factor‐like domains 2; ECM, extracellular matrix; ERK, extracellular signal‐regulated kinase;LRP1, low‐density lipoprotein receptor‐related protein 1; MK, Midkine; MMPs, matrix metalloproteinases; OA, osteoarthritis; TGF‐β1, transforming growth factor beta1; TIMP‐3, tissue inhibitor of metalloproteinases‐3; Wnt5b, Wingless‐type MMTV integration site family, member 5B.

### 5.3 NF‐κB Signaling Pathway

The NF‐κB signaling pathway is central to controlling inflammation and cell survival, with its activation being closely linked to the advancement of OA [83, 84]. LRP1 interacts with NF‐κB to modulate chondrocyte function and ECM homeostasis, primarily by inhibiting inflammation triggered by proinflammatory cytokines like TNF‐α. Yang et al. examined the role of LRP1 in modulating TNF‐α‐induced inflammation and MMP‐13 expression in chondrocytes [41]. By employing lentivirus‐mediated RNA interference to silence LRP1 expression in rat chondrocytes, they found that LRP1 deficiency heightened sensitivity to TNF‐α, leading to increased apoptosis and inflammation via activation of the NF‐κB signaling pathway. Silencing LRP1 resulted in elevated expression of apoptotic markers, including Bax and caspase‐3, while reducing levels of the antiapoptotic protein Bcl‐2. These results suggest that LRP1 exerts a protective effect on chondrocytes by counteracting TNF‐α‐induced apoptosis, thereby preserving cartilage integrity through the dual actions of inhibiting catabolic enzymes and enhancing cell survival [41]. Targeting NF‐κB inhibition under LRP1‐deficient conditions could thus provide a dual approach to prevent cartilage degeneration and promote repair.

### 5.4 TGF‐β1 Signaling Pathway

TGF‐β1 is an essential cytokine that contributes to cartilage homeostasis and skeletal growth, playing a critical role in regulating the equilibrium between anabolic and catabolic processes in joint tissues [85, 86]. Thus, understanding the interaction between TGF‐β1 signaling and LRP1 is crucial for developing targeted OA therapies [87, 88].

Recent studies have emphasized the importance of LRP1 in controlling TGF‐β1 signaling in osteoarthritic chondrocytes. Functioning as a coreceptor for TGF‐β1, LRP1 influences downstream pathways that regulate chondrocyte activity. Dennis et al. demonstrated that LRP1 directly influences TGF‐β1 signaling in chondrocytes, a process essential for maintaining cartilage integrity during skeletal differentiation. LRP1 also plays a role as a chaperone, promoting TGF‐β1 transport to the cell surface, where it influences chondrocytes and skeletal cells [78]. In a separate study, Hu et al. investigated the involvement of LRP1 in Obs, focusing on its interaction with TGF‐β1 signaling [42]. Their findings revealed consistent LRP1 expression across osteoblast populations with differing responses to vitamin D3. Notably, “high responders” exhibited increased TGF‐β1 expression compared to “low responders,” suggesting that enhanced TGF‐β1 signaling may interact with LRP1 to regulate osteoblast differentiation and bone remodeling processes [42]. These findings indicate that while LRP1 expression remains constant, its interaction with elevated TGF‐β1 could influence the osteoblast phenotype, contributing to pathological changes in subchondral bone during OA [42]. Overall, LRP1 serves as a critical regulator of TGF‐β1 signaling in osteoarthritic cartilage and subchondral bone, presenting a valuable target for the development of disease‐modifying therapies aimed at enhancing outcomes for OA patients.

### 5.5 CCN2

CCN2, commonly referred to as connective tissue growth factor, is a crucial member of the CCN protein family, playing an essential role in skeletal development [89, 90]. In cartilage biology, CCN2 promotes chondrocyte differentiation, ECM production, and endochondral ossification [28]. The interaction between CCN2 and key receptors such as LRP1 may offer novel therapeutic strategies for cartilage regeneration and OA treatment [28, 31]. Kawata et al. investigated the potential function of LRP1 as a receptor for CCN2 in chondrocytes. Their findings revealed that LRP1 facilitates the endocytic uptake of CCN2, indicating a specialized pathway for CCN2 internalization and transport within these cells [28]. Notably, CCN2 colocalized with LRP1 in recycling endosomes and the nucleus of mature chondrocytes.

Kawata et al. further investigated the involvement of LRP1 in mediating the endocytic transport and transcytosis of CCN2 in chondrocytes [31]. By employing small interfering RNA (siRNA) to suppress LRP1 expression in chondrocytes, they observed a marked decrease in the uptake of exogenous CCN2, indicating that LRP1 is crucial for the efficient endocytosis and distribution of CCN2 within cartilage tissue. Importantly, transcytosis experiments revealed that LRP1 facilitates the transport of CCN2 from the prehypertrophic layer to the hypertrophic zone of growth plate cartilage. This process likely regulates the availability of CCN2, supporting endochondral ossification and maintaining cartilage integrity. Under hypoxic conditions, which mimic the avascular environment of cartilage, LRP1 expression and CCN2 transcytosis were significantly increased, suggesting an adaptive mechanism for CCN2 delivery under stress conditions [31]. These insights present opportunities for therapeutic intervention by targeting LRP1–CCN2 interactions to enhance cartilage repair and mitigate OA progression.

### 5.6 Autophagy

Autophagy is a critical cellular process that maintains homeostasis by degrading and recycling damaged components, thus promoting cell survival under stress conditions [91, 92]. Impaired autophagy is linked to increased cell death and accelerated cartilage degeneration, contributing to OA pathogenesis. Enhancing autophagic activity in chondrocytes may, therefore, provide a promising therapeutic strategy to prevent and alleviate cartilage degeneration in OA [80].

Yan et al. investigated the effects of LRP1 deficiency on cartilage development and autophagy in a developmental dysplasia of the hip (DDH) model, a condition that increases susceptibility to OA [80]. The study used LRP1 heterozygous knockout mice (*Lrp1+/−*) and knock‐in mice carrying a missense mutation (*Lrp1* ^*R1783W*^), mimicking a human DDH mutation, to investigate the role of LRP1 in cartilage formation. The findings revealed that LRP1 deficiency significantly impaired chondrogenic differentiation of triradiate cartilage, with decreased autophagy levels and concomitant upregulation of β‐catenin. These results highlight the essential role of LRP1 in cartilage development, mediated through the regulation of autophagy [80]. Histological analysis of Lrp1‐deficient mice demonstrated premature closure of the triradiate cartilage, leading to malformations of the acetabulum and femoral head. This structural change was associated with decreased autophagy marker LC3B levels and increased p62, suggesting impaired autophagic flux in LRP1‐deficient chondrocytes. These findings highlight the therapeutic potential of targeting the LRP1–autophagy pathway to enhance cartilage health and slow the progression of OA in individuals with DDH [80].

## 6 Other Regulatory Roles of LRP1 in OA

### 6.1 MicroRNAs (miRNAs)

miRNAs are small, noncoding RNA molecules that serve critical functions in gene regulation and participate in diverse biological processes such as inflammation, cell differentiation, and apoptosis [93]. Among them, miR‐204 has shown particular promise for maintaining joint tissue homeostasis and providing chondroprotective effects by modulating cartilage metabolism (Table 4).

**TABLE 4:** The miRNA, *N*‐Glycosylation, and Proteins‐Mediated LRP1 in OA.

Name	Cell/tissue type	Mechanism	Function summary	References
Exosome‐derived proteins	Mouse cartilage	Enhanced PI3K/AKT pathway by TNF‐α preconditioning	Improves cartilage repair and mitigates OA pathology in preclinical models by regulation of LRP1 levels	[94]
IFT88	Mouse and human Chondrocytes	Regulates LRP1 endocytosis; affects extracellular protease clearance	Mutation increases aggrecanase activity; impacts ECM turnover	[69]
miR‐204	Chondrocytes, neural cells	Inhibits SP1‐LRP1 signaling; blocks neurocartilage interaction	Reduces osteoarthritis‐related pain; protects cartilage	[95]
*N*‐glycosylation proteins	KBD and normal chondrocytes	Altered glycosylation affecting PI3K/Akt signaling pathway	LRP1 are expressed at higher levels in KBD‐ induced pluripotent stem cells chondrocytes	[72]
*N*‐glycosylation proteins	Knee cartilage	Regulates ECM integrity and signal transduction	Associate with KBD pathological process, including LRP1 in KBD and OA processes	[96]

Abbreviations: ECM, extracellular matrix; IFT88, intraflagellar transport protein 88; KBD, Kashin‐Beck disease; LRP1, low‐density lipoprotein receptor‐related protein 1; OA, osteoarthritis; PI3K/Akt, phosphatidylinositol 3‐kinase (PI3K)/protein kinase B (AKT); TNF‐α, tumor necrosis factor‐alpha.

Lu et al. investigated the therapeutic implications of miR‐204 in OA and its interaction with LRP1 in modulating pain and cartilage degeneration [95]. The study revealed that miR‐204, administered via bone marrow mesenchymal stem cell (MSC)‐derived exosome mimetics (EMs), effectively reduced OA‐related pain and protected against cartilage degradation by suppressing SP1–LRP1 signaling and disrupting neurocartilage interactions. The experimental findings revealed that miR‐204 directly targets the SP1–LRP1 axis, resulting in a reduction of LRP1 expression in chondrocytes [95].

Further experiments involving cocultures of chondrocytes and Schwann cells showed that miR‐204 downregulated c‐FOS, NGF, and TRPV1 expression in Schwann cells, indicating reduced nociceptive signaling. The downregulation occurred through the inhibition of SP1, a transcription factor that binds to the LRP1 promoter, thereby decreasing LRP1 expression [95]. This study establishes the miR‐204/SP1‐LRP1 axis as a potential molecular target for managing OA‐related pain and highlights an innovative strategy for using exosome‐based miRNA delivery.

### 6.2 *N*‐Glycosylation

*N*‐glycosylation, a prevalent post‐translational modification, is crucial for numerous biological functions, such as protein folding, stability, and molecular interactions [97, 98]. In OA, *N*‐glycosylation is involved in maintaining cellular homeostasis and chondrocyte differentiation. Abnormal *N*‐glycosylation has been linked to cartilage‐associated disorders, including OA and Kashin–Beck disease (KBD), where it contributes to compromised ECM integrity and heightened vulnerability to cartilage damage [72, 96].

Lyu et al. performed a comparative analysis of the *N*‐glycosylation patterns in knee cartilage from patients with KBD and OA [96]. The study identified 278 different *N*‐glycosylation sites across 187 *N*‐glycoproteins, with significant differences between KBD and OA samples. Interestingly, LRP1 was identified as a key protein with a distinctive *N*‐glycosylation site (Asn‐4125) present exclusively in KBD samples, indicating that this modification may impact the role of LRP1 in ECM integrity and chondrocyte homeostasis [96]. In addition, Wang et al. expanded on this work by investigating *N*‐glycosylation patterns in chondrocytes derived from iPSCs from individuals with KBD and healthy controls [72]. Their findings demonstrated elevated *N*‐glycosylation levels of LRP1 in KBD‐derived chondrocytes compared to normal counterparts, which correlated with aberrant cell signaling and impaired ECM regulation. Bioinformatics analysis revealed that LRP1 exhibited differential expression linked to the phosphatidylinositol 3‐kinase (PI3K)/protein kinase B (Akt) signaling pathway, highlighting a potential link between LRP1 glycosylation and altered cellular functions that may contribute to cartilage degeneration in KBD. These results highlight the value of glycoproteomic profiling in identifying effective treatments for cartilage pathologies.

### 6.3 Exosomes

Exosomes, a class of extracellular vehicles (EVs) measuring 30–150 nm in size, have gained significant attention for their role in mediating cellular communication and their therapeutic applications [99]. In the context of OA, exosomes derived from MSCs have shown promise in mitigating cartilage degradation, alleviating synovial inflammation, and regulating immune responses [100]. Given their cell‐free nature, exosome‐based therapies present a compelling approach for OA treatment due to their cell‐free nature, which minimizes the risks of immune rejection and tumorigenicity commonly associated with direct stem cell therapies [101].

Wu et al. explored the impact of exosomes derived from infrapatellar fat pad (IPFP)‐MSCs preconditioned with TNF‐α on OA [94]. Their study revealed that TNF‐α preconditioning markedly enhanced exosome secretion from IPFP‐MSCs, primarily via activation of the PI3K/AKT signaling pathway. This pathway activation resulted in the upregulation of the autophagy‐related protein ATG16L1, which promoted increased exosome production [94]. Additionally, proteomic analysis of exosomes derived from TNF‐α‐preconditioned IPFP‐MSCs (IPFP‐MSC‐EXOs^TNF‐α^) revealed a significant enrichment of LRP1, a protein known for its chondroprotective properties. LRP1 is essential for cartilage homeostasis, mediating the endocytosis and breakdown of ECM‐degrading enzymes such as MMPs and ADAMTSs. Further studies indicated that the increased LRP1 expression observed in OA chondrocytes treated with IPFP‐MSC‐EXOs^TNF‐α^ was likely attributed to the uptake of these LRP1‐enriched exosomes. The increased presence of LRP1 promoted the degradation of ECM‐degrading enzymes, indirectly enhancing ECM stability and contributing to cartilage integrity [94].

Immunohistochemical analysis confirmed higher levels of LRP1 and aggrecan in cartilage tissue treated with IPFP‐MSC‐EXOs^TNF‐α^, suggesting that these exosomes enhance the anabolic processes necessary for cartilage repair. This underscores the therapeutic potential of exosomal LRP1 and introduces an innovative strategy to enhance the efficacy of MSC‐derived exosomes, offering a promising avenue for advancing clinical treatments for OA [94].

## 7 LRP1 as a Promising Target for Subchondral Bone Remodeling in OA

Subchondral bone is essential for maintaining joint health, serving as a structural foundation for the articular cartilage above and dissipating mechanical stress [102, 103]. Emerging research has shed light on the involvement of LRP1 in modulating subchondral bone remodeling during the progression of OA [103]. For instance, Zhang et al. investigated changes in gene expression within the subchondral bone in an early experimental OA model [79]. Their gene expression profiling revealed a significant downregulation of LRP1 at 1 and 2 weeks postsurgery, indicating that LRP1 may play a crucial role in the initial stages of OA development. This reduction in LRP1 expression in subchondral bone could lead to compromised bone quality and subsequent cartilage deterioration, highlighting its vital role in preserving subchondral bone integrity during the progression of OA [79].

In another study, Hu et al. investigated LRP1 expression in Obs derived from the subchondral bone of patients undergoing knee replacement surgery [42]. The study identified two distinct populations of osteoblasts based on their response to vitamin D3 stimulation: high responders and low responders. They found that LRP1 expression was not significantly altered between these two groups under basal conditions or in response to vitamin D3, suggesting that LRP1 expression remains stable in Obs [42]. Conversely, genes associated with bone metabolism, including TGF‐β1 and Wnt5b, displayed varied expression patterns, underscoring the intricate signaling networks governing subchondral bone remodeling. These results indicate that LRP1 plays a pivotal role in subchondral bone remodeling, especially in the early phases of OA development [42]. The stable expression of LRP1 in osteoblasts, despite changes in other signaling molecules, indicates that LRP1 may serve as a regulatory anchor in maintaining bone homeostasis. On the other hand, its downregulation in early OA points to its involvement in initiating pathological changes in subchondral bone.

## 8 Conclusions and Future Directions

LRP1 has become a key focus in understanding the pathogenesis of OA, particularly its influence on cartilage integrity, inflammatory pathways, and ECM regulation [28, 30, 33]. The impairment of this endocytic pathway, largely due to increased shedding of LRP1 in osteoarthritic cartilage, results in elevated levels of these catabolic enzymes and accelerates cartilage destruction [38]. Restoration of LRP1 function has been shown to reduce ECM degradation and maintain cartilage integrity, suggesting that targeting LRP1 could be an effective therapeutic approach for OA [38, 39]. Another significant aspect of LRP1 in OA may influence susceptibility to OA by modulating Wnt, NF‐κB pathway activity, and *N*‐glycosylation, underscoring the potential for personalized treatment strategies based on genetic profiles [41, 77, 96].

While in vitro studies and preclinical models suggest that restoring LRP1 function can reduce ECM degradation and maintain cartilage integrity, there is a lack of robust in vivo studies confirming these findings over the long term. Additionally, the influence of LRP1 on signaling pathways like Wnt/β‐catenin, NF‐κB, and *N*‐glycosylation is evident, but the precise mechanisms of these interactions remain unclear, particularly their stage‐specific effects during OA progression. The development of engineered exosomes as a delivery system for LRP1‐related therapeutics represents another promising direction in OA research [94]. Exosomes are effective vehicles for delivering therapeutic molecules, including proteins, RNAs, and drugs, to target tissues. In the context of OA, for exosome‐based therapies, optimizing production, cargo loading, and targeting specificity is essential to ensure the efficient delivery of LRP1‐related molecules to chondrocytes and subchondral bone. In addition, engineered exosomes can be functionalized with surface markers to enhance their targeting efficiency. For example, exosomes derived from MSCs could be loaded with LRP1 mRNA or proteins and delivered directly to chondrocytes, promoting cartilage repair and reducing inflammation [94].

Beyond exosome‐based approaches, gene editing tools like CRISPR‐Cas9 offer an innovative strategy to regulate LRP1 expression in chondrocytes [80]. By targeting promoter regions or editing regulatory elements, CRISPR‐Cas9 can directly upregulate LRP1 expression, offering precise control over its activity. To achieve this, delivery systems such as lipid nanoparticles and adeno‐associated viruses (AAV) are being optimized to ensure the specific transport of CRISPR‐Cas9 components to chondrocytes within the joint. Furthermore, the use of joint‐specific promoters could enhance the precision of LRP1 modulation, minimizing off‐target effects and promoting localized therapeutic action. Despite these advancements, significant challenges remain, particularly in achieving safe and efficient delivery of gene‐editing components while reducing potential off‐target effects [104, 105]. In parallel, small molecule modulators present another promising approach to regulate LRP1 activity [61, 74]. Such molecules could pharmacologically enhance the ability of LRP1 to mediate endocytosis or prevent its shedding, restoring its function in osteoarthritic cartilage. Compared to inhibitors targeting IL‐1β and TNF‐α, LRP1 not only modulates inflammatory pathways but also plays a pivotal role in ECM regulation and cartilage integrity. This dual role enables LRP1 to provide a more comprehensive therapeutic approach, modulating multiple pathways (e.g., Wnt/β‐catenin, NF‐κB) while enhancing chondrocyte survival and maintaining tissue homeostasis. In contrast, MMP and ADAMTS inhibitors focus solely on reducing ECM degradation, lacking the capacity to restore cartilage integrity. LRP1, through its endocytic functions, not only diminishes the activity of these catabolic enzymes but also supports cartilage repair mechanisms. This unique profile makes LRP1 an attractive therapeutic target with stage‐specific applications.

At the early stages of OA, therapies targeting LRP1 could prioritize preventing cartilage degradation by enhancing the endocytic clearance of ECM‐degrading enzymes and regulating key signaling pathways. This approach aims to slow disease progression and preserve cartilage homeostasis. In advanced OA, LRP1‐based therapies could complement regenerative strategies, such as CRISPR‐Cas9‐mediated gene editing or exosome‐engineered delivery of LRP1‐enhancing molecules. These combined approaches may facilitate cartilage repair and improve overall joint function, offering hope for both disease modification and tissue regeneration.

The application of advanced single‐cell RNA sequencing and spatial transcriptomics technologies offers significant potential to analyze LRP1 expression across various cell types within the joint, shedding light on how these expression patterns evolve throughout disease progression [106, 107]. By mapping LRP1 expression at the single‐cell level, researchers can identify specific cell populations that may be particularly important for maintaining cartilage integrity or driving disease progression. In addition, advanced imaging, such as live‐cell microscopy, is being used to monitor the delivery and activity of these therapies in real time. This information could be used to develop more targeted therapies that focus on modulating LRP1 activity in specific cell types, thereby improving treatment efficacy and minimizing side effects.

In summary, recent advances in elucidating the role of LRP1 in OA underscore its significance as a key regulator of cartilage homeostasis and a promising therapeutic target. Harnessing innovative treatment strategies could pave the way for disease‐modifying therapies that go beyond symptom relief to restore joint function and enhance the quality of life for OA patients.

## Author Contributions

**Yuangang Wu** and **Kaibo Sun** wrote the manuscript. **Mingyang Li**, **Yang Yang**, **Yuan Liu**, **Limin Wu** and **Yang Ding** edited the manuscript and designed the figures. **Yi Zeng** and **Bin Shen** contributed to the manuscript design. All authors read and approved the final manuscript.

## Conflicts of Interest

The authors declare no conflicts of interest.
